# Observations on a Disease Consequent to Transplanting Teeth

**Published:** 1789

**Authors:** George Spence

**Affiliations:** Dentist to His Majesty


II. *Obſervations on a Diſeaſe conſequent to tranſplanting Teeth. By Mr.* George Spence, *Dentiſt to His Majeſty.*

THE diſeaſe, of which I mean to treat, is that which ariſes after the tranſplanting of teeth, and which has, as yet, only appeared (as Mr. Hunter expreſſes it) in "conſequence of "two living parts being brought into contact;" for I have never ſeen or heard that the diſeaſe has followed the inſertion of a dead tooth: but although, in ſeveral inſtances, ſuch a diſeaſe has ariſen in conſequence of tranſplanting a living one, yet theſe are few when compared with the number of teeth tranſplanted.

Tranſplanting teeth is by no means a new operation, or reſtricted to this country. We find it mentioned by Ambroſe Paré; and it is practiſed at this time by the moſt eminent dentiſts upon the Continent. Neither the ancients nor the moderns, however, have diſtinguiſhed any diſeaſe as being peculiar to the tranſplanting what, for diſtinction's ſake, we will call a living tooth, notwithſtanding they have expreffly treated of tranfplanting the tooth of one living perfon into the head of another.

In fyftematic writings we find complaints of this kind claffed according to the fituation of the parts affected, or a fuppofed predifponent caufe; hence we find them generically arranged among the dental, gingival, alveolar, or maxillary pains, ulcers, fiftulas, &c. and thefe, again, reduced to different fpecies of rheumatic, fcorbutic, fcrophulous, or venereal affections, which are always confidered as latent in the conftitution, and not as being implanted with the tooth.

The fymptoms and progrefs of the difeafe, fo far as I have been able to collect from the inftances in which it has occurred to me, have been as follows:—About five or fix weeks after the tooth has been tranfplanted, and apparently well fixed, upon the patient's taking cold, the gum has fwelled, become red and painful, and then receded from the tooth; ulceration has taken place, the teeth have grown loofe, and the difcharge has become very fœtid: if, before this period, the difeafe has not been checked by medical treatment, the tooth has dropped out, fymptoms of hectic fever have enfued, and blotches have appeared upon the fkin. In all the cafes, however, that have fallen under my obfervation the difeafe has terminated favourably, and given way to medicine, excepting in one inftance, in which the hectic fymptoms deftroyed the patient.

An exfoliation of the alveolar procefs generally takes place in this difeafe; but whether it arifes from the teeth having been tranfplanted, or from the great irritation confequent to the operation, or from any other unfortunate circumftance connected with the teeth, I muft acknowledge myfelf at a lofs to determine.

To this concife general view of the appearances and progrefs of the difeafe I will now add a more particular account of the fymptoms, as they occurred in the different perfons who were the unhappy fubjects of it, and will alfo take notice of the methods of treatment. To fome of the patients the complaint gave but little trouble, while in others it excited very unpleafant fymptoms; and indeed to all it occafioned no fmall alarm.

In relating the cafes I fhall arrange them more according to the method of treatment than to the order of time in which they occurred. The firft of thefe cafes was under the direction of my father; the others have occurred to me, in the courſe of twelve years, in my own practice.

### CASE I.

A lady had a tooth tranſplanted, and five weeks after the operation, having danced and caught cold, ſhe had a fever for ſix weeks; at the end of which time the diſeaſe appeared in the gum. The bicuſpis, which had been tranſplanted, and the tooth next to it, were removed; the alveolar proceſs exfoliated; the ulceration was obſtinate; and a node-like ſwelling appeared upon the leg: but the whole eventually gave way to ſea bathing and the internal uſe of ſea water. This caſe, as well as ſome of the others which I ſhall mention, has been publiſhed by Mr. Hunter in his work on the venereal diſeaſe.

### CASE II.

A young lady, who had had the middle inciſor tranſplanted, caught cold, while in the country, about the ſixth week after the operation, and the gum became tumid, red, and painful. Expecting the complaint to go off as a common cold, ſhe did not apply for advice till her return to town, which was in about ten days after. Ulceration had then taken place, and extended to one of the other teeth; and from the foreign tooth the gum had receded, and the ulcerated ſurface diſcharged a fœtid matter. The bark was now directed to be uſed freely, and two or three glaſſes of red port to be drank at dinner. The diſeaſed part was waſhed with a ſtrong decoction of bark, and a pledget dipped in it was applied to the gums and teeth. Mr. Hunter ſaw the diſeaſe in this ſtate, and adviſed the quantity of bark to be increaſed, the uſe of wine to be continued, and the three teeth to be removed. The ſockets exfoliated, and in about four weeks the patient was perfectly well, and has had no relapſe.

### CASE III.

A young lady having had one of the inciſores tranſplanted, it fixed, and did well till about five weeks after the operation; at which time ſhe danced, and caught cold. In a day or two after this the gum ſwelled, grew painful, and then became ulcerated. The ulceration was proceeding as in the other caſe; but, by the advice of two ſurgeons of eminence, the tooth having been extracted, and the bark given freely, the gum healed, and the young lady was perfectly reſtored in a few weeks.

CASE

### CASE IV.

A young lady had a tooth tranſplanted, and during the firſt five weeks the ſame favourable appearances enſued that have generally attended this operation. The gum then began to ulcerate, and the ulceration increaſed. A ſurgeon, who was conſulted, adviſed the uſe of mercury; but it was not had recourſe to, and nothing more was done than extracting the tooth. After this the gum healed as faſt as any common ulcer uſually does.

### CASE V.

A young lady had two inciſores tranſplanted, and, ſeemingly, with ſucceſs till about ſix weeks after the operation, when an ulceration took place, with the uſual ſymptoms, and attended with great pain. The ulceration, however, ſpread very ſlowly.

Her family ſurgeon, who was conſulted, thought the diſeaſe venereal, and that it aroſe from contagion conveyed by the tranſplanted tooth. Another ſurgeon was called in, who objected to a mercurial courſe, and deſired that the bark might be uſed. During the uſe of the latter remedy the diſeaſe was apparently getting getting better, though not ſo faſt as the ſurgeon of the family wiſhed; he therefore had recourſe to mercury, and in a few weeks the ulceration was ſtopped, and the patient appeared to be well. The diſeaſe, however, returned again ſeveral times, but was finally removed by a perſeverance in the uſe of mercury.

It ſhould be obſerved, that from the ſame girl, out of whoſe head the implanted tooth was taken, another lady had a tooth the day before, and that in that caſe no ulceration followed. The girl was examined before the operation, and again when the ulceration had begun in the caſe I have been relating, by three ſurgeons, who pronounced her free from any diſeaſe.

### CASE VI.

A gentleman had two inciſores tranſplanted, and in about five weeks after the operation ulceration took place, and was attended with all the uſual ſymptoms. He was adviſed to take the Peruvian bark freely, and to apply a ſtrong decoction of it to the ulcerated gum, the diſcharge from which was very acrid and fœtid. This mode of treatment was purſued during three or four weeks, and both the tranſplanted teeth were kept in. The gum, however, on being healed, was found to have receded a little. This gentleman had the appearance of being of a ſcrophulous habit.

### CASE VII.

A gentleman, who was evidently of a ſcrophulous habit, had three teeth tranſplanted, and about five weeks after the operation took cold by falling aſleep in the open air, when warm and fatigued. Ulceration came on, and proceeded in the manner uſual in theſe caſes. At firſt, recourſe was had to the bark; but a phyſician, who was called in, deeming the caſe venereal, put him under a courſe of mercury. There were blotches on the ſkin, and his eyes were inflamed. He recovered in the courſe of a few weeks, and his teeth were preſerved. I ſaw him many months after, and he was then perfectly well.

### CASE VIII.

A young lady, who had an inciſor tranſplanted, caught cold in the country about a month afterwards, and ulceration took place in the gum, and ſpread conſiderably. I recommended a lotion of tincture of bark and myrrh, and a pledget dipped in it to be applied to the part. A ſurgeon, who ſaw her ſoon after, adviſed a ſolution of corroſive ſublimate to be applied to the gum; but this, as it gave her pain, was ſoon diſcontinued. Another ſurgeon was then conſulted, who did not think the caſe venereal, and who only directed her to waſh her mouth with barley water and tincture of myrrh. As the complaint increaſed, recourſe was had to the Peruvian bark and myrrh, but without any perceptible good effect. The pain and tenderneſs of the parts prevented her taking food and reſt, ſo that her health was ſoon greatly impaired. In this ſtate ſhe became a patient of the late Sir William Watſon, who has given an account of the caſe in the Medical Tranſactions *; as hath likewiſe Mr. Hunter in his Treatiſe on the Venereal Diſeaſe †.

The diſeaſe grew better while the patient was under a courſe of mercury, but ſhe died conſumptive. Of this caſe Mr. Hunter remarks, that the quantity of mercury taken was not ſufficient to cure ſuch chancres in other parts, or

* Vol. III. page 325.—See alſo Vol. VII. of this work, page 216.

† Page 391.

venereal fores on the fkin; and even this quantity did not appear to affect the conftitution, but went off by the bowels. He expreffly fays, that, upon the whole, he fhould think it not venereal; and even Sir William Watfon, who, with others, upon firft feeing the difeafe in its worft ftage, thought it venereal, allows that great difficulties (and to him even infurmountable ones) prefented themfelves in the inveftigation of the caufe; and he fays of the cure, that the mercury never took to the patient's gums, or falivary ducts, in the leaft, an event which was much to be dreaded. From hence it appears that mercury, if it had taken in the leaft to the gums, or falivary glands, would have been, in his opinion, a very dangerous remedy; but as it never affected either, it can hardly be faid, in this cafe, to have produced any fpecific effect.

I fhall now mention a cafe in which mercury was given, in confequence of venereal infection in the natural way, recently after tranfplanting. The patient was a gentleman, who had the two front incifores tranfplanted, and the operation feemed to have fucceeded; but a few weeks after he caught the lues venerea, and went under a mercurial courfe. A confiderable ulceration took place in the gums not only of the the tranſplanted, but alſo of the two lateral teeth, in conſequence of which they all four came out.

On the appearance of any new diſeaſe, or of any diſeaſe from a new cauſe, great circumſpection is neceſſary in deciding upon its origin. The fact itſelf, as proved by the caſes I have related, is, that from a living tooth being put into the jaw of another perſon, in ſome inſtances, a diſeaſe hath been produced, ſimilar, in ſome of its ſymptoms, to the lues venerea, and which has given way either to the removal of the tooth, or to the uſe of medicines.

Thus in four of the caſes we have ſeen that it gave way after the removal of the tooth, without any mercury being adminiſtered:

In two others it yielded to the removal of the tooth, and to the uſe of mercury:

In one of the caſes it yielded to the uſe of bark, without the removal of the tooth:

And in another caſe it gave way to mercury, without the removal of the tooth.

From this review of the caſes it ſeems clearly to appear that nothing is ſo likely to ſtop the progreſs of the diſeaſe as the removal of the tooth. The other indications of cure will conſiſt in allaying the local irritation, and in ſtrengthening thening the conftitution. Mercury, as we have feen, was had recourfe to in fome of the cafes; but I am inclined to think that in thofe it acted rather as an alterative than as a fpecific, for in neither of the cafes, confidered as a fpecific, did it appear to have any claim to the cure.

The theories that have been held out, relative to this affection, have been founded either on an opinion that contagion is conveyed in thefe cafes by the tooth, or on the idea of a predifpofition to the difeafe in the fyftem of the patient, which is called forth and excited by the irritation of the tooth.

On the fuppofition of its originating from contagion, it has been deemed venereal; while, on the other hand, by thofe who afcribe it to a predifponent caufe, it has been rather thought fcrophulous. Medical men, who have feen only one inftance of the difeafe, have been of the former, while thofe who have attended many are of the latter opinion. Without reftricting myfelf to the names affigned to it by either, I fhall give my reafons for thinking that it arifes from a predifpofing caufe in the patient, and of courfe is rendered eventually precarious, as well as all other operations: at the fame time I muft obferve, that it bears as good an afpect as any, and and indeed a better one than moſt others, if the number of ſucceſsful caſes be compared with that of the unſucceſsful.

As this ſubject has been ſo ably treated by Mr. Hunter, I am happy in availing myſelf of his opinion; and ſhall expreſs mine, ſo far as we agree in opinion, in the language he has made uſe of: poſitively confirming, at the ſame time, his and the other accounts given of the young perſons from whom the teeth were taken, that they had not, in appearance, the venereal diſeaſe in any of its ſtages, but were perfectly well.

Firſt, then, I think, with Mr. Hunter, that it does not ariſe from a venereal taint, becauſe ſome patients, whoſe caſes were ſimilar to the others, recovered without the uſe of medicine of any kind, and ſeveral without the ſpecific medicine, mercury.

Secondly, As the perſons from whom the teeth were taken had no appearance of the diſeaſe, it ſeems ſtrange that it ſhould break out in the receivers, and not in the givers.

Thirdly, I conſider it as impoſſible for parts to have the power of contaminating which have not themſelves the diſeaſed action.

Fourthly, The parts contaminating were never known to have been contaminated themſelves.

Laftly, The argument that arifes from the difeafe having, in feveral inftances, given way to mercury, will have little force when it is confidered that the fame remedy has been ufed with great fuccefs in India in cafes of hepatitis, and by Dr. Hamilton in peripneumony, &c. and is employed by many practitioners in cutaneous diforders, and likewife in fcrophulous affections; in the latter of which the pilula Plummeri, with the external application of mercurius corrofivus ruber to the ulcers, has had the happieft effect.

It is Mr. Hunter's opinion, that if a fore be produced by other means in the found part of a perfon affected with the lues, that fore is not venereal, nor the matter poifonous, although formed from the blood *. If, therefore, (fuppofing this opinion to be well founded) the wound made by extracting a tooth be confidered as a recent fore, nothing conveyed by that tooth can be fuppofed to be contagious, efpecially when we confider that the perfon's mouth, gums, and teeth, are to all appearance found; fo that, upon the whole, I am perfectly of opinion with Mr. Hunter, that, whether we confider the ori-

* See his Treatife on the Venereal Difeafe, page 290.

gin, gin, progrefs, or cure of the difeafe, there is no good reafon to think it venereal.

I am perfuaded that this difeafe more probably arifes from a predifponent caufe, operating in confequence of irritation, for the following reafons:

Firft, In feveral of the inftances a removal of the irritating caufe prevented farther mifchief; for the bark and wine that were prefcribed feemed to have no farther fhare in the cure than, as tonics, by preventing and correcting a debility which would have aggravated the difeafe.

Secondly, The difeafe generally makes its appearance after the threads have been removed, and the tooth is left to fupport itfelf; and this I think will account, in a great meafure, for the equable periods at which the difeafe has arifen; and the circumftance of its generally beginning after fome violent exercife, fuch as dancing or taking cold, (which tend to produce an inflammatory affection that will naturally be determined to this weak part) ferves confiderably to confirm my opinion, that, in perfons predifpofed to this difeafe, the difturbance given to the teeth, together with the weaknefs and irritability of the parts, are to be confidered as the remote caufes of this complaint.

 Thirdly,

Thirdly, There is a diſeaſe that appears very ſimilar to this, which ariſes naturally, and very often in ſituations where ſcrophula is almoſt endemic; a low ſituation, a bad climate, and a poor diet, generally diſpoſe to it, and any accidental occurrence calls it forth. In the fens of Lincolnſhire, where ſcrophula often affects the lower claſs of people, it appears very commonly in the mouths of children and young people at teething, and ſhedding the teeth, but moſt violently at the latter; at which time, if the looſe teeth are not removed, the gum becomes tumid, red, and painful, diſcharges a fœtid matter, and is attended with hectic ſymptoms, which ſometimes terminate fatally.

In theſe caſes the ulceration of the gums is often attended with ſcrophulous ulcers, in the cure of which a better diet, the uſe of tonics, of mercury with opiates, and occaſionally of ſea bathing, have frequently been found ſucceſsful: but in a caſe of this kind, which fell under my own obſervation, the ſymptoms differed ſo little from thoſe of the diſeaſe produced by the tranſplanting of teeth, that the complaint was treated in the ſame manner, and gave way as that diſeaſe uſually does. The ſubject of this caſe was a young gentleman in London,

London, about four years of age, whofe four incifores of the under jaw became loofe. A confiderable ulceration of the gums took place, accompanied with a very fœtid difcharge, and the alveolar procefs was a little expofed.

When I examined the teeth and gums, I was of opinion that nothing could be fuccefsfully done while the teeth were in. The father of the child objected to their being drawn, and wifhed the cure might be attempted without; but after trying various remedies the patient got worfe.

In a fhort time I faw him again, and then it was agreed that the teeth fhould be taken out. I accordingly drew them: the bark was then given freely, and in a few weeks the four fockets exfoliated, and the patient got well. He was of a fcrophulous habit, and was fent to the fea for the purpofe of bathing, which proved of great fervice to him.

This cafe clearly proves that where there is a predifpofition to this difeafe it may be, and is, brought into action by an original tooth as well as by one taken from another perfon.

I have now endeavoured to lay before the reader all that has fallen under my obfervation relative to the fymptoms, progrefs, and cure of a difcaſe which I cannot conſider as a new one, notwithſtanding one of its proximate cauſes has not been frequently attended to, or been often known to produce it. I do not ſpeak the language of a perſon whoſe experience has placed his opinion beyond the reach of doubt; but, after laying all circumſtances together, I cannot help thinking it of as much conſequence, if not more, to the ſucceſs of tranſplanting teeth, that the conſtitution of the patient ſhould be free from hereditary or latent diſeaſe, as that the tooth received ſhould be ſound.

*Pall Mall,*

July 7th, 1789.

