# Errata

**Published:** 1789

**Authors:** 


— 259, l. 1, for *four years of age*, read *twelve years of age*.

